# Corrosion threshold data of metallic materials in various operating environment of offshore wind turbine parts (tower, foundation, and nacelle/gearbox)

**DOI:** 10.1016/j.dib.2019.104207

**Published:** 2019-07-03

**Authors:** J.I. Ahuir-Torres, S. Simandjuntak, N. Bausch, A. Farrar, S. Webb, A. Nash, B. Thomas, J. Muna, C. Jonsson, D. Matthew

**Affiliations:** a School of Mechanical and Design Engineering, United Kingdom; b School of Energy and Electronic Engineering, University of Portsmouth, Anglesea Road, Portsmouth, PO1 3DJ, United Kingdom; c Avonwood Developments Ltd, Bournemouth, BH21 7ND, United Kingdom; d Avanti Communications, London, EC4V 6EB, United Kingdom

**Keywords:** Offshore, Wind turbines, Detection, Monitoring, Corrosion sensor, Electrochemical analysis, OCP, ZRA, EIS and PPC

## Abstract

This paper outlines corrosion thresholds for different environmental conditions of metallic materials commonly used in the tower, foundation, and nacelle/gearbox of an offshore wind turbine. These threshold values were derived from laboratory corrosion testing employing electrochemical analysis techniques, using the media/solvents that are representative to the operating environment of those wind turbine parts, such as seawater, grease, oils/lubricants, or their combination, at room temperature and at 328K. These values can provide an indication when general/local corrosion or protective film/surface damages have occurred. They can thus be utilised for detecting and monitoring corrosion at certain locations in the wind turbine structure. The presented data have been verified and validated to ensure their repeatability and reliability by means of numerous laboratory tests in accordance to the relevant engineering test standards and an extensive literature/published data review.

**Table:** Specifications table

Subject area	Chemistry
More specific subject area	Corrosion of Metals
Type of data	Tables
How data was acquired	Electrochemical analysis methods: Open Circuit Potential (OCP), Zero Resistance Ammeter (ZRA), Electrochemical Impedance Spectroscopy (EIS) and PotentiodynamicPolarisation Curve (PPC) Facilities: Potentio/galvanostat, model GillAC, made by ACM Instruments Software: Gill AC serial no 600
Data format	Raw Data and Analysed
Experimental factors	In accordance to the recommended and relevant international test standards [1], [2], [3], [4], [5]
Experimental features	Test samples: Metallic materials include low carbon structural steel S235 and S355, stainless steels SS316L and SS430, Aluminium alloys AA1010, AA3103, AA5052 and AA6061. Corrosion testing: OCP utilised a two electrodes cell. ZRA, EIS and PPC utilised a three electrodes cell. The test samples were corroded artificially by PPC. OCP, ZRA and EIS were conducted on non-corroded and corroded test samples at room temperature (RT) and at 328K. Environment/Solutions/Media: • Substitute ocean water of pH = 8.2 (referred to as ‘Seawater’) for RT testing (ASTM-D1141) [5]; • A commercial engine semi-solid lubricant containing corrosion inhibitor (referred to as ‘Grease’) of a measured pH = 5.2 for RT testing (ASTM D6547) [3]; • Dissolution of 30% (*Wt/Wt*) Grease and 70% (*Wt/Wt*) Seawater of a measured pH = 4.3 for RT testing; and of pH = 6.8 for testing at 328K (ASTM D665) [4], • A commercial oil (Poly-Alpha- Olefin) of a measured pH = 8.8 for RT testing; and of pH = 8.6 for testing at 328K (ASTM D6547) [3].
Data source location	School of Mechanical and Design Engineering (SMDE), University of Portsmouth, Hampshire, United Kingdom
Data accessibility	The data is with this article
Related research article	J.I. Ahuir-Torres, N.Bausch, A. Farrar, S. Webb, S. Simandjuntak, a. Nash, B. Thomas, J. Muna, C.Jonsson, D. Mathew, Benchmarking parameters for remote electrochemical corrosion detection and monitoring of offshore wind turbine structures, Wind Energy, 22–6 (2019), 857–876.

**Table:** 

**Value of the data** • The data generated from laboratory testing following the known/internal standards are threshold ranges or values that can be used to validate and indicate when general/local corrosion or protective film/surface damages on metallic materials on various offshore wind turbine structures in their typical environments. • The Nyquist and Bode diagrams could be useful for other researchers fitting such data to equivalent circuits in order to gain insights into the actual mechanism of corrosion. • Plant operators, inspection/maintenance companies, WT design industries will benefit from one source database with an open access privilege to assist the work in this field or in the structural health monitoring technology development. • The data can be integrated into an operating system such as a SCADA-like system for remote detection and monitoring of corrosion/surface damages through the implementation of a Real Time Remote Sensing (RTRS) technology. • The data could help in furthering the understanding of corrosion failure mechanisms of the selected metallic materials used in offshore WT parts, which can be used to consolidate and/or optimise the design of the relevant parts with respect to their material selection and operating conditions.

## 1 Data

The investigated metallic materials commonly used in the foundation, tower and nacelle/gearbox of an offshore WT with their typical environments are listed in Table 1.

**Table 1:** Investigatedmetallic materials commonly used in the offshore WT tower, foundation and nacelle/gearbox and their typical operating conditions/environment.

WT Parts	Environment	Metallic Materials/Alloys
Foundation, Tower	Seawater	Stainless steel (SS) 316L Structural steel (S) 355 Aluminium Alloys (AA) 3103, AA5052
Nacelle/Gearbox	Semi solid lubricants (Grease) with added corrosion inhibitor	SS430 S235 AA1010 AA6061
	Oil/lubricant (e.g. Poly-Alpha-Olefin)	
	Mixed environment (Seawater/Grease/Oil)	

The Open Circuit Potential (OCP), Zero Resistance Ammeter (ZRA), Electrochemical Impedance Spectroscopy (EIS) and PotentiodynamicPolarisation Curve (PPC) are the electrochemical analysis techniques utilised in conjunction with the conducted corrosion testing. Table 2 highlights the characteristics of each of these techniques and the relationships between their relevant corrosion parameters and outputs. The nomenclatures of these parameters are outlined in Table 3.

**Table 2:** Electrochemical analysis techniques.

Techniques	Characteristics	Equations	Outputs
Open Circuit Potential (OCP) [7], [8]	• Non-destructive • Passive • Detect corrosion • Inform type of corrosion (passive film damage, localised and uniform/general corrosion)	• Ecel=Eo+(R∗T)(n∗F)∗ln([Prod]P[React]R)	Potential (*E*), Units: Voltage (*V*)
Zero Resistance Ammeter (ZRA) [7], [8]	• Non-destructive • Passive • Detect corrosion • Inform type of corrosion (passive film damage, localised and uniform/general corrosion) • •Calculate corrosion rate, *C.R.*	• IR.M.S=∑i=1n|In|N • C.R.=IR.M.S∗MF∗d∗n	Current density (*I*), Units: Amps/square centimetres (*A/cm*^*2*^)
Electrochemical Impedance Spectroscopy (EIS) [8]	• Non-destructive • Active, • Uses Alternating Current (AC) • Detect corrosion • Inform type of corrosion (localised and uniform/general corrosion) • Indirect analysis on corrosion mechanisms e.g. diffusion, passivation or activation • Indirect analysis on characteristics of the corrosion products or processes e.g. diffusion, adsorption-desorption or water absorption	• Z(f)=Eo∗sin(2∗π∗f∗t)Io∗sin(2∗π∗f∗t+θ) • ⇒θ=0R(f)=Z(f)=EoIo • ⇒θ=maxC(f)=12∗π∗fmax∗R(fmax) • L=εr∗εo∗AC(f)	Impedance (*Z*), Units: Ohm per square centimetres (*Ω/cm*^*2*^)
Potentio-dynamic Polarisation Curves (PPC) [7]	• Destructive • Active • Uses Direct Current (DC) • Detect corrosion • Inform type of corrosion (passive film damage, localised and uniform/general corrosion) • Indirect analysis on corrosion mechanisms e.g. diffusion, passivation or activation • Determine corrosion rate, *C.R.*	• Eapplied−Ecorr=βclog(IcIcorr)+βalogIaIcorr • Icorr=βc∗βa2.303∗(βc+βa)∗Rp	Potential (*E*), Unit: Voltage (*V*) Current density (*I*), Unit: Amps/square centimetres (*A/cm*^*2*^)

**Table 3:** Nomenclatures.

Symbol	Significance
*V/V*	Volume/Volume
*Wt/Wt*	Weight/Weight
*E*_*cel*_	Cell potential
*E*^*o*^	Reference potential
*K*	Gas constant
*T*	Temperature
*n*	Number of the transferred electrons in the corrosion reaction
*F*	Faraday constant
*[Prod]*	Molar concentration of the products
*[React]*	Molar concentration of the reactants
^*P*^	Stoichiometric factor of the products
^*R*^	Stoichiometric factor of the reactants
*I*_*n*_	Current density for each readings
*N*	Number of readings
*I*_*R.M.S*_	Root Mean Square of the current density
*C.R*.	Corrosion rate
*M*	Molar mass
*d*	Density of the material
*Z*_*(f)*_	Impedance according to the frequency
*E*_*o*_	Amplitude of the potential
*I*_*o*_	Current density amplitude
*f*	Frequency
*t*	time
*θ*	Angle of phase
*R*_*(f)*_	Resistance
*C*_*(f)*_	Capacitance
*f*_*max*_	Frequency at maximum angle of phase
*R*_*(fmax)*_	Resistance at maximum angle of phase
*L*	Thickness of the corrosion product or process
*ε*_*r*_	Relative permittivity
*ε*_*o*_	Permittivity of the vacuum
*A*	Area
*E*_*applied*_	Applied potential
*E*_*corr*_	Corrosion potential
*β*_*c*_	Cathodic slope
*β*_*a*_	Anodic slope
*I*_*c*_	Cathodic current density
*I*_*a*_	Anodic current density
*I*_*corr*_	Corrosion current density
*R*_*p*_	Polarization resistance
*E*	Potential
*I*	Current density
*R*	Resistance
*C*	Capacitance
*D.A.*	Data Acquisition
*t*_*Total*_	Total time of the experiments
Δ*f*	Frequencies range
Δ*V(R.M.S)*	Root mean square amplitude of the potential
*S.R*	Sweep Rate
*E*_*ini*_	Initial potential
*E*_*ocp*_	Potential to open circuit
*E*_*final*_	Final potential
*E*_*Ref*_	Potential of the reference electrode
*I*_*lim*_	Limit current density
*Z*_*real*_	Real Impedance
*Z*_*imag*_	Imaginary Impedance
*Z*_*mod*_	Impedance Modulus

The corrosion threshold ranges or values for various different environmental conditions of the investigated alloys are therefore essentially of the four mentioned electrochemical analysis techniques’ parameters. These values tabulated in Table 4, Table 5 are compiled with regards to the types of corrosion i.e. uniform/general or localised corrosion. The table also includes the selected references that are used to verify the presented data. The extensive literature/published data review indicated a large variability in the methods/procedures of testing and data generation. Therefore, those references containing only work performed in accordance to the international standards were considered in the review and for the data verification. In addition, Table 6 represents PPC analysed data (βa and βc) of the metallic materials from the corrosion testing conducted at different environments. These parameters can be used to evaluate the corrosion rate, C.R. (their relationship is shown in Table 2), thus for life prediction.

**Table 4:** Corrosion threshold ranges or values for different environment conditions in association with the uniform/general corrosion of the commonly used metallic materials in foundation, tower and nacelle of an offshore WT.

Material/Alloy	WT Parts	General Corrosion					*Notes
		Environment	Corrosion Data				
			*E* (V)	*I* (A/cm^2^)	*R* (Ω*cm^2^)	*C*(F/cm^2^)	
SS316L	Foundation, Tower	Seawater at RT and pH = 8.2	>-0.140, 0.400<	<1.500*10^−7^	>6.245*10^4^	<1.739*10^−5^	[10], [11], [12]/Oxidised Surface
					>6.928*10^5^	<4.149*10^−5^	[10], [11], [12]/Bare Surface
SS430	Nacelle/Gearbox	Grease at RT and pH = 5.2	>-0.040, 3.000<	<3.452*10^−9^	<4.255*10^6^	<1.266*10^−11^	a/Lubricant
					<2.258*10^8^	>1.619*10^−8^	a/Bare Surface
		Grease & Seawater (30:70 wt/wt) at RT and pH = 4.3	>-0.063, 3.000<	<3.005*10^−8^	<1.023*10^6^	>3.020*10^−11^	a/Lubricant
					>7.000*10^7^	<1.721*10^−8^	a/Bare Surface
		Grease & Seawater (30:70 wt/wt) at 328K and pH = 6.8	>-0.180, 1.102<	<1.261*10^−7^	<2.820*10^4^	>1.260*10^−10^	a/Lubricant
					<1.590*10^6^	<3.920*10^−6^	a/Bare Surface
		Oil at RT and pH = 8.8	<-0.237, 1.375<	<2.484*10^−10^	<4.597*10^6^	>8.805*10^−12^	a/Lubricant
					>3.810*10^8^	>6.938*10^−9^	a/Bare Surface
		Oil at 328K and pH = 8.6	>-0.042, 3.000<	<1.521*10^−9^	<6.036*10^4^	>6.721*10^−11^	a/Lubricant
					>1.880*10^6^	<4.755*10^−6^	a/Bare Surface
S235		Grease at RT and pH = 5.2	>-0.060, 3.000<	<3.354*10^−9^	<2.121*10^6^	<7.834*10^−12^	a/Lubricant
					>2.990*10^8^	>7.441*10^−8^	a/Bare Surface
		Grease & Seawater at RT and pH = 4.3	>-0.160, 3.000<	<2.506*10^−9^	>1.314*10^6^	>3.426*10^−11^	a/Lubricant
					>7.640*10^7^	<1.885*10^−8^	a/Bare Surface
		Grease & Seawater at 328K and pH = 6.8	>-0.220, 0.990<	<1.249*10^−7^	<1.270*10^4^	<5.664*10^−11^	a/Lubricant
					<6.720*10^5^	<6.637*10^−7^	a/Bare Surface
		Oil at RT and pH = 8.8	<1.400, 3.000<	<3.615*10^−10^	<6.234*10^6^	<9.074*10^−12^	a/Lubricant
					<6.810*10^8^	>1.262*10^−8^	a/Bare Surface
		Oil at 328K and pH = 8.6	>0.070, 3.000<	<8.254*10^−10^	<3.700*10^4^	>1.079*10^−10^	a/Lubricant
					>4.490*10^6^	>9.727*10^−6^	a/Bare Surface
AA1010		Grease at RT and pH = 5.2	>-0.506, 3.000<	<3.546*10^−9^	<4.968*10^6^	>3.795*10^−11^	a/Lubricant
					>3.360*10^8^	<1.822*10^−8^	a/Bare Surface
		Grease & Seawater at RT and pH = 4.3	>-0.760, −0.400<	<2.163*10^−8^	>2.178*10^6^	<2.235*10^−11^	a/Lubricant
					>3.530*10^7^	<9.919*10^−9^	a/Bare Surface
		Grease & Seawater at 328K and pH = 6.8	>-0.700, 3.000<	<1.443*10^−7^	>5.476*10^4^	<1.142*10^−10^	a/Lubricant
					>6.300*10^5^	<2.067*10^−6^	a/Bare Surface
		Oil at RT and pH = 8.8	>-0.190, 0.600<	<1.038*10^−10^	>7.362*10^6^	<9.515*10^−12^	a/Lubricant
					>4.855*10^8^	<2.830*10^−9^	a/Bare Surface
		Oil at 328K and pH = 8.6	>-0.250, 3.000<	<2.940*10^−10^	<4.388*10^4^	<9.422*10^−11^	a/Lubricant
					>2.896*10^6^	<2.861*10^−6^	a/Bare Surface
AA6061		Grease at RT and pH = 5.2	>-0.290, 3.000<	<1.190*10^−10^	<4.633*10^6^	<1.609*10^−11^	a/Lubricant
					>4.633*10^8^	<1.346*10^−8^	a/Bare Surface
		Grease & Seawater at RT and pH = 4.3	>-0.546, 0.840<	<1.000*10^−8^	>2.150*10^6^	<2.405*10^−11^	a/Lubricant
					>4.434*10^7^	<1.129*10^−8^	a/Bare Surface
		Grease & Seawater at 328K and pH = 6.8	>-0.741, 0.230<	<1.678*10^−7^	<2.462*10^4^	>2.593*10^−10^	a/Lubricant
					<7.741*10^5^	<4.345*10^−7^	a/Bare Surface
		Oil at RT and pH = 8.8	>0.718, 3.000<	<4.383*10^−10^	>1.370*10^7^	>1.549*10^−11^	a/Lubricant
					>4.580*10^8^	>9.389*10^−9^	a/Bare Surface
		Oil at 328K and pH = 8.6	>-0.129, 3.000<	<5.669*10^−9^	<7.402*10^6^	<3.908*10^−11^	a/Lubricant
					>4.250*10^6^	<1.205*10^−6^	a/Bare Surface

*Notes:

Numbers indicate references of the reviewed literatures/documents that were used to verify the data.

a Indicates data validated by in-house (repetitive) testing.

**Table 5:** Corrosion threshold ranges or values for different environment conditions in association with the localised corrosion and passive film damage of the commonly used metallic materials in foundation, tower and nacelle of an offshore W.

Material/Alloy	WT Parts	Localised Corrosion					*Notes
		Environment	Corrosion Data				
			*E* (V)	*I* (A/cm^2^)	*R* (Ω*cm^2^)	*C*(F/cm^2^)	
SS316L	Foundation, Tower	Seawater at RT and pH = 8.2	<-0.140, 0.400<	>2.500*10^−7^	<6.245*10^4^	>1.739*10^−5^	[10], [11], [12]/Oxidised Surface
					<6.928*10^5^	>4.149*10^−5^	[10], [11], [12]/Bare Surface
S355			<-0.680, −0.650<	>1.456*10^−5^	<1.420*10^3^	>7.906*10^−4^	a/Oxidised Surface
					<2.660*10^2^	<3.597*10^−4^	a/Bare Surface
					≤9.475*10^3^	≥3.722*10^−4^	a/Diffusion
AA5052			<-0.650, −0.570<	>4.560*10^−6^	≥4.890*10^3^	>7.353*10^−6^	a/Oxidised Surface
					>3.671*10^3^	>1.751*10^−5^	a/Bare Surface
					≤1.538*10^4^	≥4.736*10^−4^	a/Diffusion
			<-0.960, −0.750<	–	–	–	[13]
AA3103			<-0.650, −0.630<	>1.560*10^−6^	>4.200*10^3^	>8.340*10^−6^	a/Oxidised Surface
					>2.756*10^3^	≥1.500*10^−5^	a/Bare Surface
					≤2.538*10^4^	≥1.423*10^−4^	a/Diffusion
			<-1.060, −0.510<	–	–	–	[14], [15]
SS430	Nacelle/Gearbox	Grease at RT and pH = 5.2	<-0.040, 3.000<	>3.452*10^−9^	>4.255*10^6^	>1.266*10^−11^	a/Lubricant
					>2.258*10^8^	<1.619*10^−8^	a/Bare Surface
		Grease & Seawater at RT and pH = 4.3	<-0.063, 3.000<	>3.005*10^−8^	>1.023*10^6^	<3.020*10^−11^	a/Lubricant
					<7.000*10^7^	>1.721*10^−8^	a/Bare Surface
		Grease & Seawater at 328K and pH = 6.8	<-0.180, 1.102<	>1.261*10^−7^	>2.820*10^4^	<1.260*10^−10^	a/Lubricant
					>1.590*10^6^	>3.920*10^−6^	a/Bare Surface
		Oil at RT and pH = 8.8	>-0.237, 1.375<	>2.484*10^−10^	>4.597*10^6^	<8.805*10^−12^	a/Lubricant
					<3.810*10^8^	<6.938*10^−9^	a/Bare Surface
		Oil at 328K and pH = 8.6	<-0.042, 3.000<	>1.521*10^−9^	>6.036*10^4^	<6.721*10^−11^	a/Lubricant
					<1.880*10^6^	>4.755*10^−6^	a/Bare Surface
S235		Grease at RT and pH = 5.2	<-0.060, 3.000<	>3.354*10^−9^	>2.121*10^6^	>7.834*10^−12^	a/Lubricant
					<2.990*10^8^	<7.441*10^−8^	a/Bare Surface
		Grease & Seawater at RT and pH = 4.3	<-0.160, 3.000<	>2.506*10^−9^	<1.314*10^6^	<3.426*10^−11^	a/Lubricant
					<7.640*10^7^	>1.885*10^−8^	a/Bare Surface
		Grease & Seawater at 328K and pH = 6.8	<-0.220, 0.990<	>1.249*10^−7^	>1.270*10^4^	>5.664*10^−11^	a/Lubricant
					>6.720*10^5^	>6.637*10^−7^	a/Bare Surface
		Oil at RT and pH = 8.8	<1.400, 3.000<	>3.615*10^−10^	>6.234*10^6^	>9.074*10^−12^	a/Lubricant
					>6.810*10^8^	<1.262*10^−8^	a/Bare Surface
		Oil at 328K and pH = 8.6	<0.070, 3.000<	>8.254*10^−10^	>3.700*10^4^	<1.079*10^−10^	a/Lubricant
					<4.490*10^6^	<9.727*10^−6^	a/Bare Surface
AA1010		Grease at RT and pH = 5.2	<-0.506, 3.000<	>3.546*10^−9^	>4.968*10^6^	<3.795*10^−11^	a/Lubricant
					<3.360*10^8^	>1.822*10^−8^	a/Bare Surface
		Grease & Seawater at RT and pH = 4.3	<-0.760, −0.400<	>2.163*10^−8^	<2.178*10^6^	>2.235*10^−11^	a/Lubricant
					<3.530*10^7^	>9.919*10^−9^	a/Bare Surface
		Grease & Seawater at 328K and pH = 6.8	<-0.700, 3.000<	>1.443*10^−7^	<5.476*10^4^	>1.142*10^−10^	a/Lubricant
					<6.300*10^5^	>2.067*10^−6^	a/Bare Surface
		Oil at RT and pH = 8.8	<-0.190, 0.600<	>1.038*10^−10^	<7.362*10^6^	>9.515*10^−12^	a/Lubricant
					<4.855*10^8^	>2.830*10^−9^	a/Bare Surface
		Oil at 328K and pH = 8.6	<-0.250, 3.000<	>2.940*10^−10^	>4.388*10^4^	>9.422*10^−11^	a/Lubricant
					<2.896*10^6^	>2.861*10^−6^	a/Bare Surface
AA6061		Grease at RT and pH = 5.2	<-0.290, 3.000<	>1.190*10^−10^	>4.633*10^6^	>1.609*10^−11^	a/Lubricant
					<4.633*10^8^	>1.346*10^−8^	a/Bare Surface
		Grease & Seawater at RT and pH = 4.3	<-0.546, 0.840<	>1.000*10^−8^	<2.150*10^6^	>2.405*10^−11^	a/Lubricant
					<4.434*10^7^	>1.129*10^−8^	a/Bare Surface
		Grease & Seawater at 328K and pH = 6.8	<-0.741, 0.230<	>1.678*10^−7^	>2.462*10^4^	<2.593*10^−10^	a/Lubricant
					>7.741*10^5^	>4.345*10^−7^	a/Bare Surface
		Oil at RT and pH = 8.8	<0.718, 3.000<	>4.383*10^−10^	<1.370*10^7^	<1.549*10^−11^	a/Lubricant
					<4.580*10^8^	<9.389*10^−9^	a/Bare Surface
		Oil at 328K and pH = 8.6	<-0.129, 3.000<	>5.669*10^−9^	>7.402*10^6^	>3.908*10^−11^	a/Lubricant
					<4.250*10^6^	>1.205*10^−6^	a/Bare Surface

*Notes:

Numbers indicate the references of the reviewed literatures/documents that were used to verify the data.

a Indicates data validated by in-house (repetitive) testing.

**Table 6:** PPC data for the corrosion rate calculation.

Material/Alloy	WT Parts	Environment	*β*_*c*_ (V/decade)	β_a_ (V/decade)	^∗^Notes
SS316L	Foundation, Tower	Seawater at RT and pH = 8.2	0.097	0.296	a
S355			0.034	0.163	a
AA5052			0.585	0.072	a
AA3103			0.055	0.044	a
SS430	Nacelle/Gearbox	Grease at RT and pH = 5.2	0.540	1.256	a
		Grease & Seawater at RT and pH = 4.3	0.658	1.549	a
		Grease & Seawater at 328K and pH = 6.8	0.303	1.102	a
		Oil at RT and pH = 8.8	0.385	0.992	a
		Oil at 328K and pH = 8.6	0.101	0.827	a
S235		Grease at RT and pH = 5.2	0.150	1.250	a
		Grease & Seawater at RT and pH = 4.3	0.648	1.500	a
		Grease & Seawater at 328K and pH = 6.8	0.189	1.235	a
		Oil at RT and pH = 8.8	0.404	0.870	a
		Oil at 328K and pH = 8.6	0.062	0.565	a
AA1010		Grease at RT and pH = 5.2	0.221	0.616	a
		Grease & Seawater at RT and pH = 4.3	0.648	1.500	a
		Grease & Seawater at 328K and pH = 6.8	0.189	1.235	a
		Oil at RT and pH = 8.8	–	1.769	a
		Oil at 328K and pH = 8.6	0.300	0.610	a
AA6061		Grease at RT and pH = 5.2	0.060	0.585	a
		Grease & Seawater at RT and pH = 4.3	0.496	0.773	a
		Grease & Seawater at 328K and pH = 6.8	0.215	1.013	a
		Oil at RT and pH = 8.8	0.337	1.059	a
		Oil at 328K and pH = 8.6	0.044	0.520	a

*Note:

a Indicates data validated by in-house (repetitive) testing.

## 2 Experimental design, materials, and methods

Test samples or coupons of an approximately 2.0cm × 2.0cm × 0.3cm were prepared from the metallic materials listed in Table 1. They were polished using a 1200-grit paper, subsequently in a dissolution comprised of 10% (*V/V*) colloidal silica gel (0.06 μm colloidal silica gel) and 90% (*V/V*) distilled water. Following the polishing stage, the metallic samples were washed and cleaned with a commercial detergent and fresh water, then with distilled water and by isopropanol, then dried up using hot air (ASTM E3-11) [6]. A minimum of 0.5 cm^2^ polished surface area is needed to guarantee a sufficient exposure/contact during the corrosion testing.

The set-up and conditions for the corrosion testing in a substitute ocean water environment (from this point onward is referred to as ‘Seawater’) are in accordance with ASTM D1141 [5]. Meanwhile, the corrosion testing to simulate the conditions and environments in the nacelle/gearbox follows the ASTM D6547 [3] recommendation when using semi solid lubricants with added corrosion inhibitor (from this point onward is referred to as ‘Grease’), the ASTM D665 [4] when using a mixture of 30% (*Wt/Wt*) Grease and 70% (*Wt/Wt*) Seawater, and the ASTM D6547 [3] for testing using oils at RT and at 328K.

Electrochemical corrosion testing was performed using a potentio/galvanostat (*GillAC, ACM Instruments*) that was controlled by software *Gill AC serial n*^*o*^ *600*. OCP utilises a two electrodes cell, namely a working and a reference electrode. ZRA, EIS and PPC added a second working (a sacrificial) or counter electrode to construct a three electrodes cell system. Silver/silver chloride potassium chloride saturated (Ag/AgCl Sat. KCl) was used as the reference electrodes and graphite rods as the second working or counter electrodes. The test sample was the other working electrode.

**Fig. 1:**
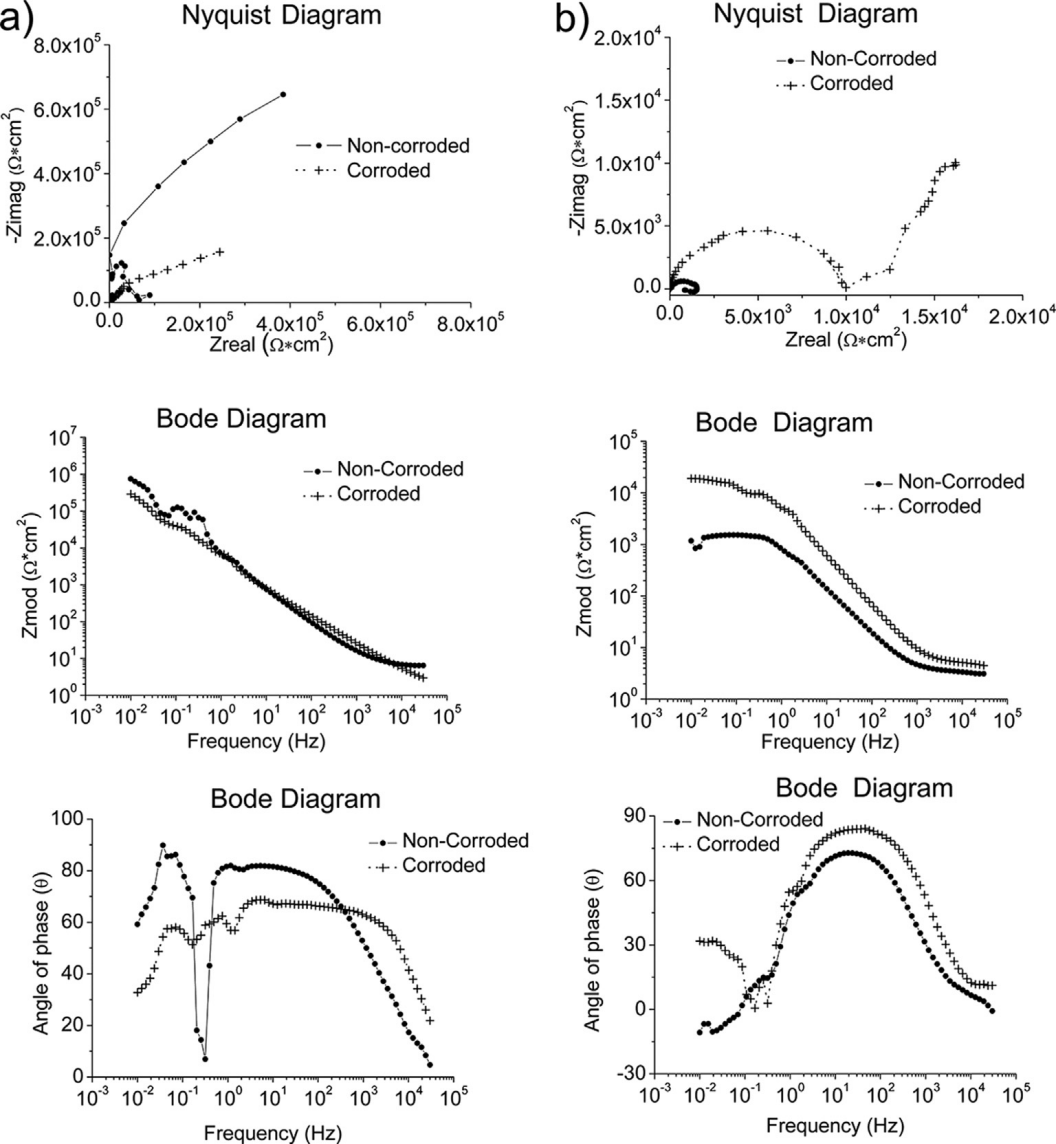

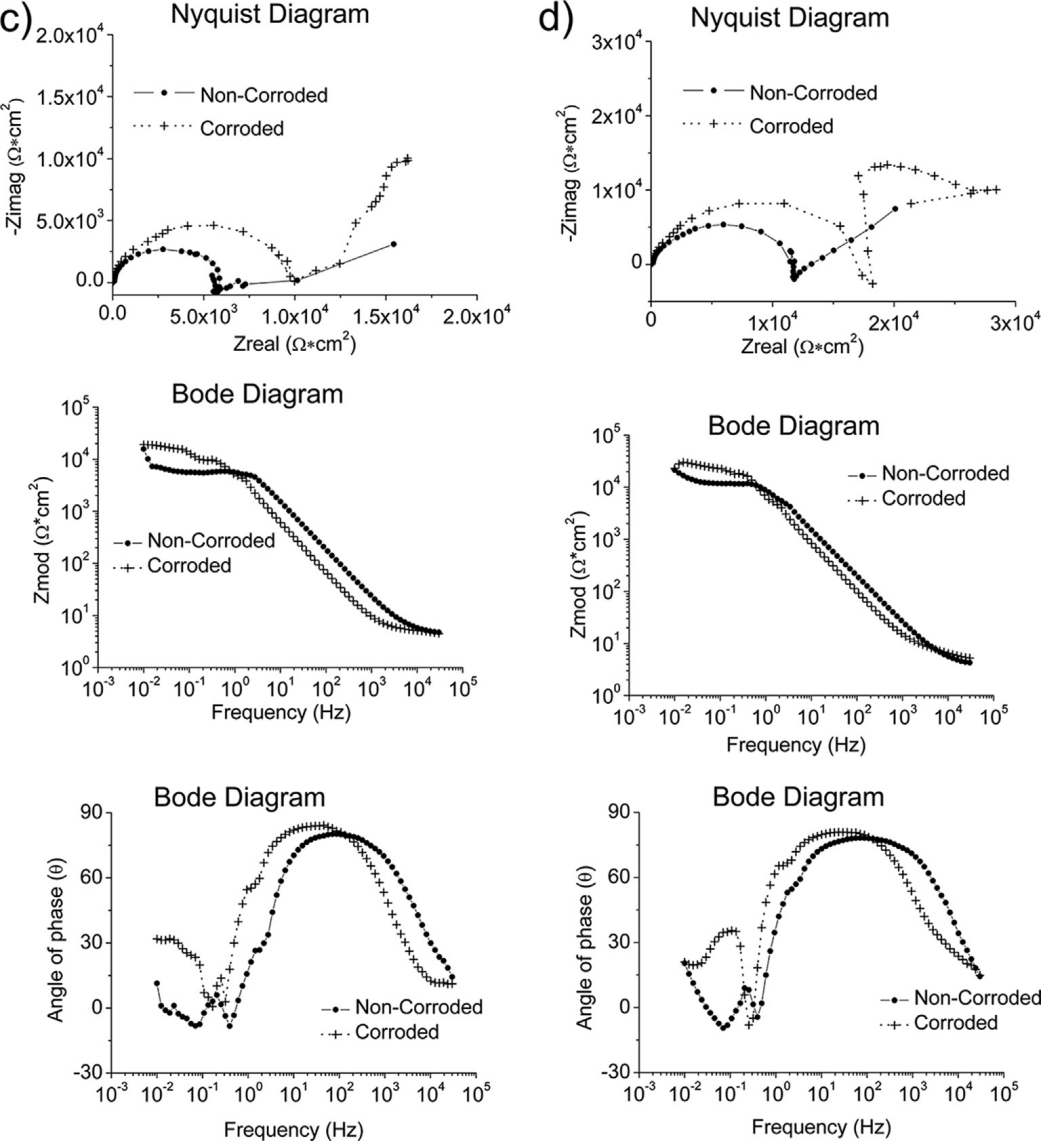
The Bode and Nyquist diagrams of non-corroded and corroded materials immersed in artificial seawater: a) SS316L, b) S355, c) AA5052 and d) AA3103.

**Fig. 2:**
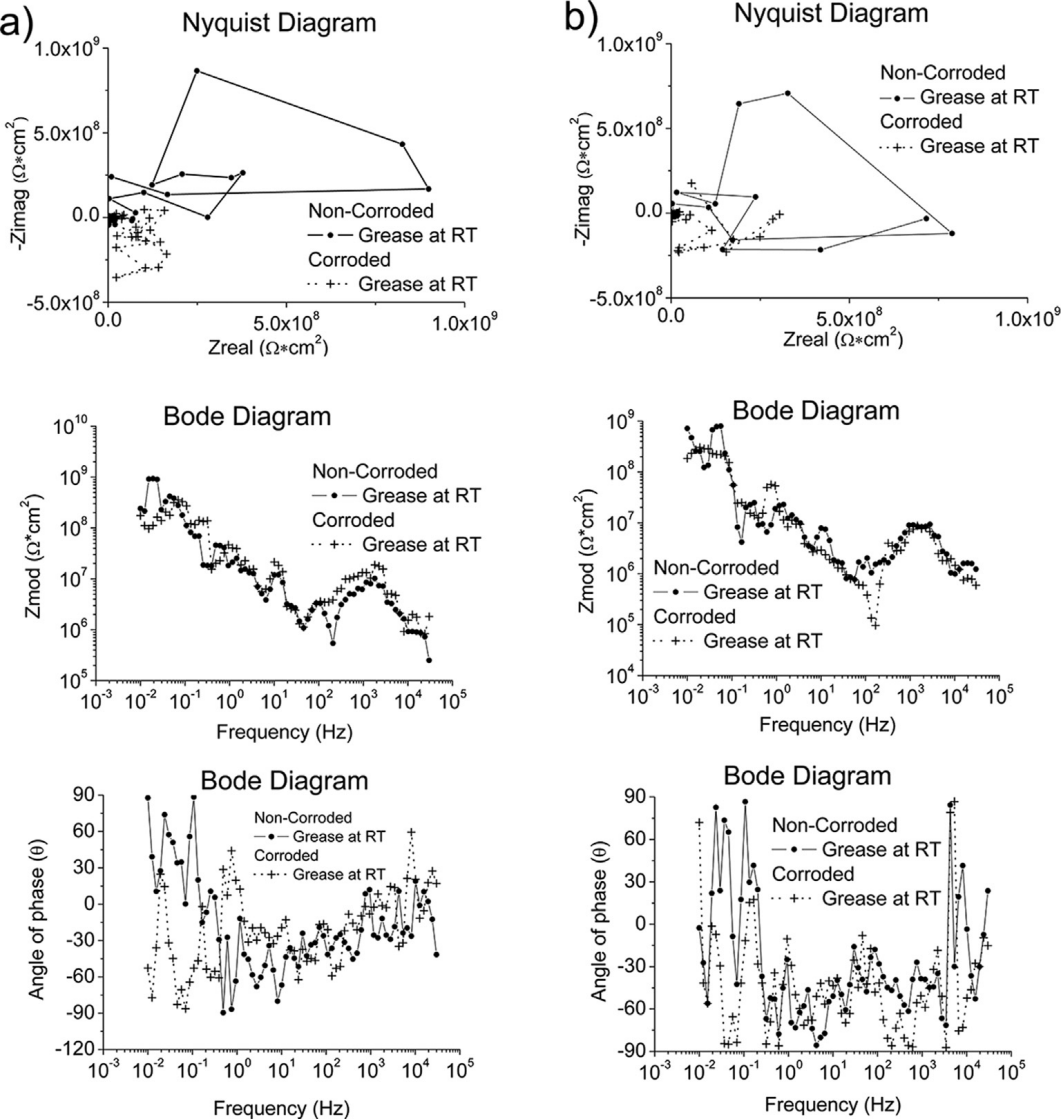

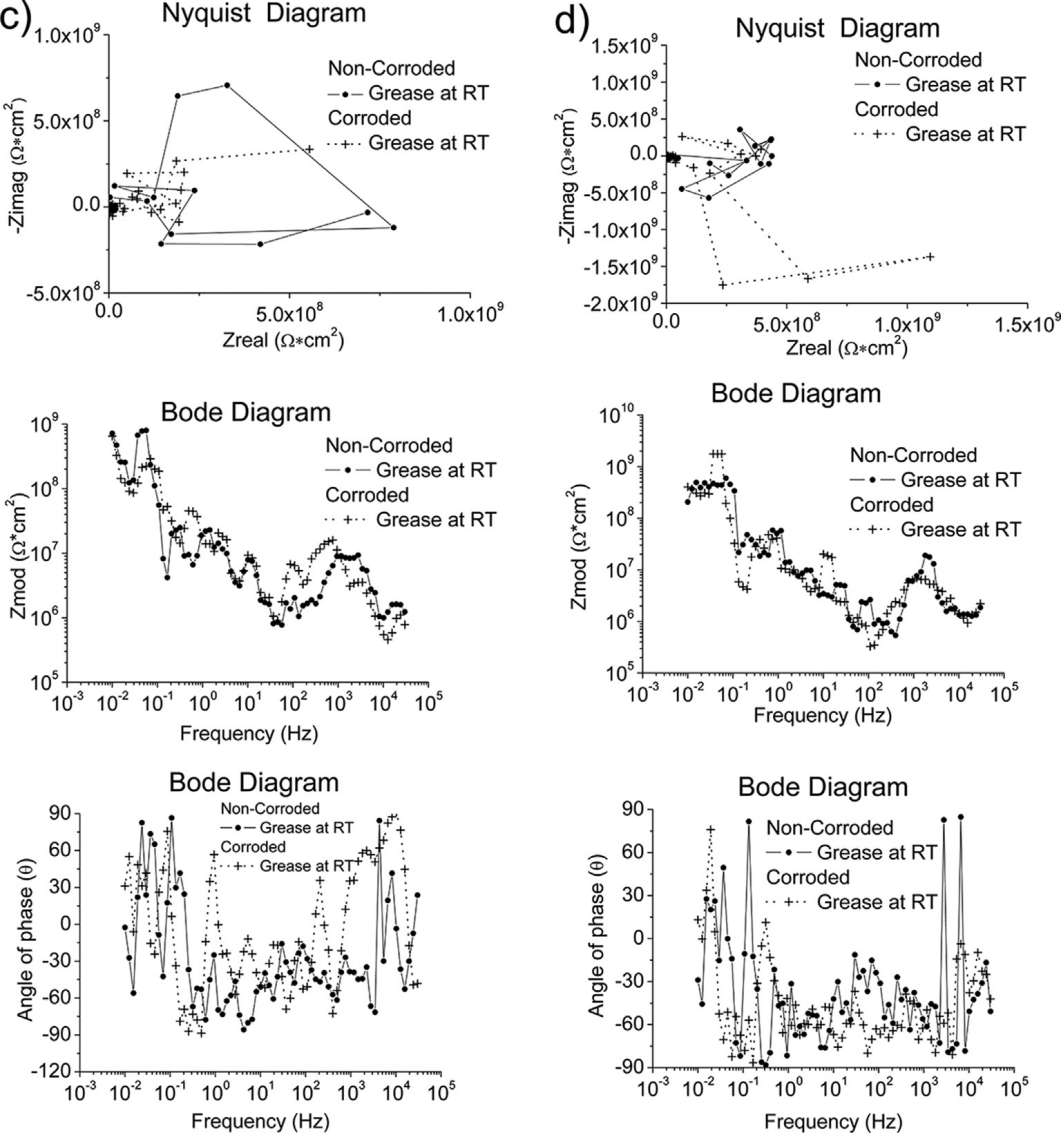
The Bode and Nyquist diagrams of non-corroded and corroded materials subjected to grease at room temperature: a) SS430, b) S235, c) AA1010 and d) AA6061.

**Fig. 3:**
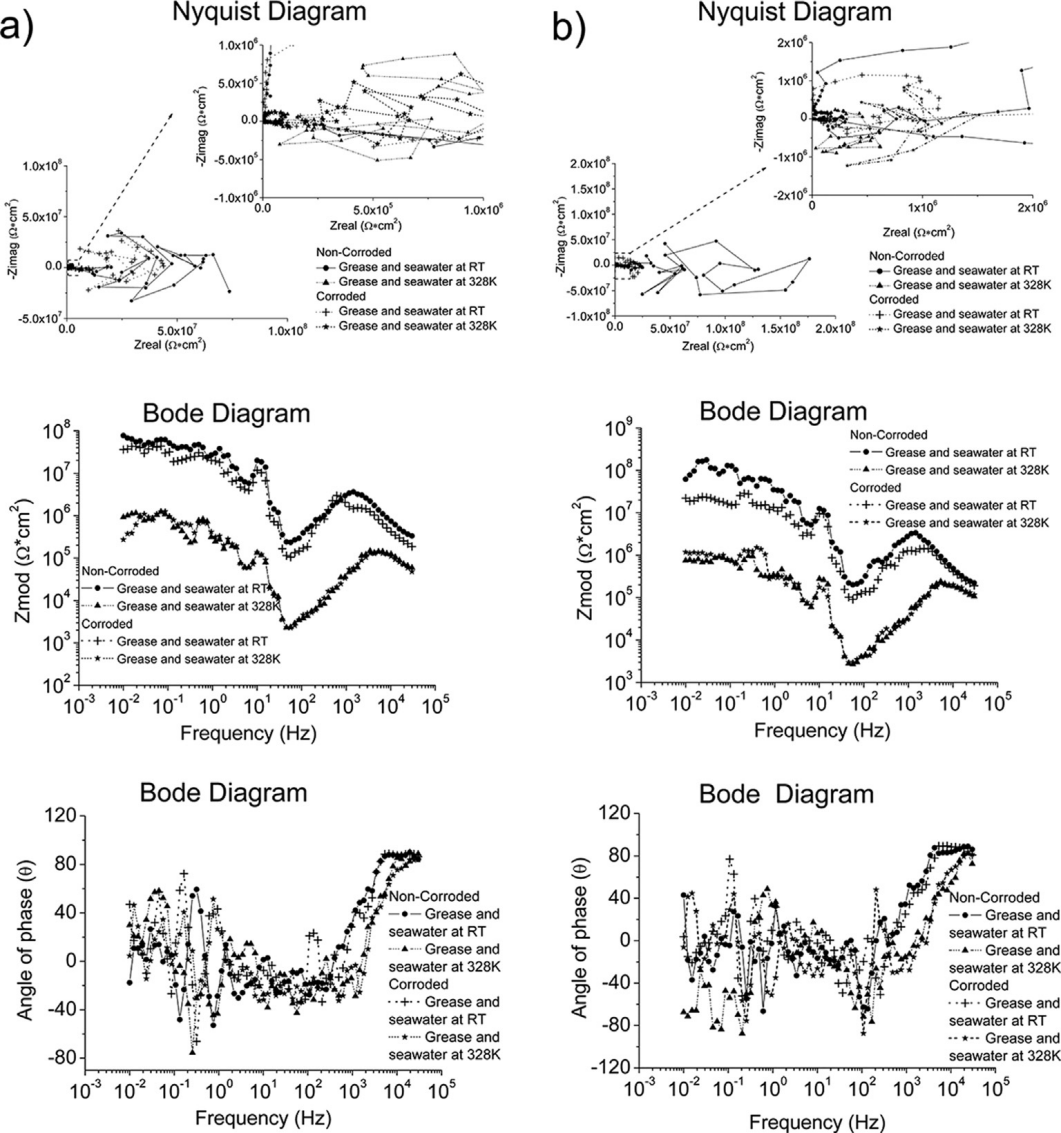

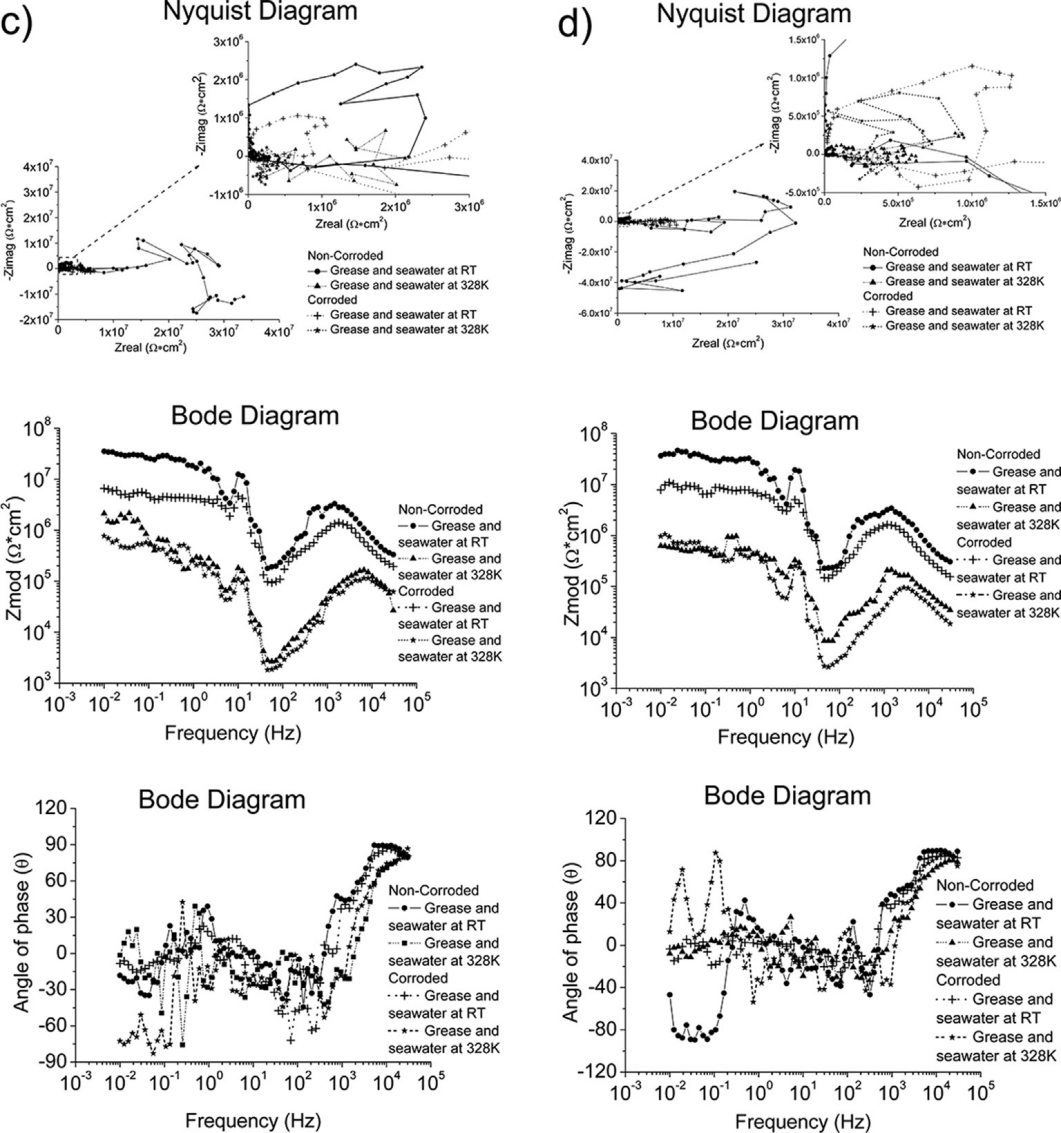
The Bode and Nyquist diagram of non-corroded and corroded materials subjected to grease & seawater at room temperature and at 328K: a) SS430, b) S235, c) AA1010 and d) AA6061. Note: The zoom-in area from the Nyquist diagram is shown in the insert plot.

**Fig. 4:**
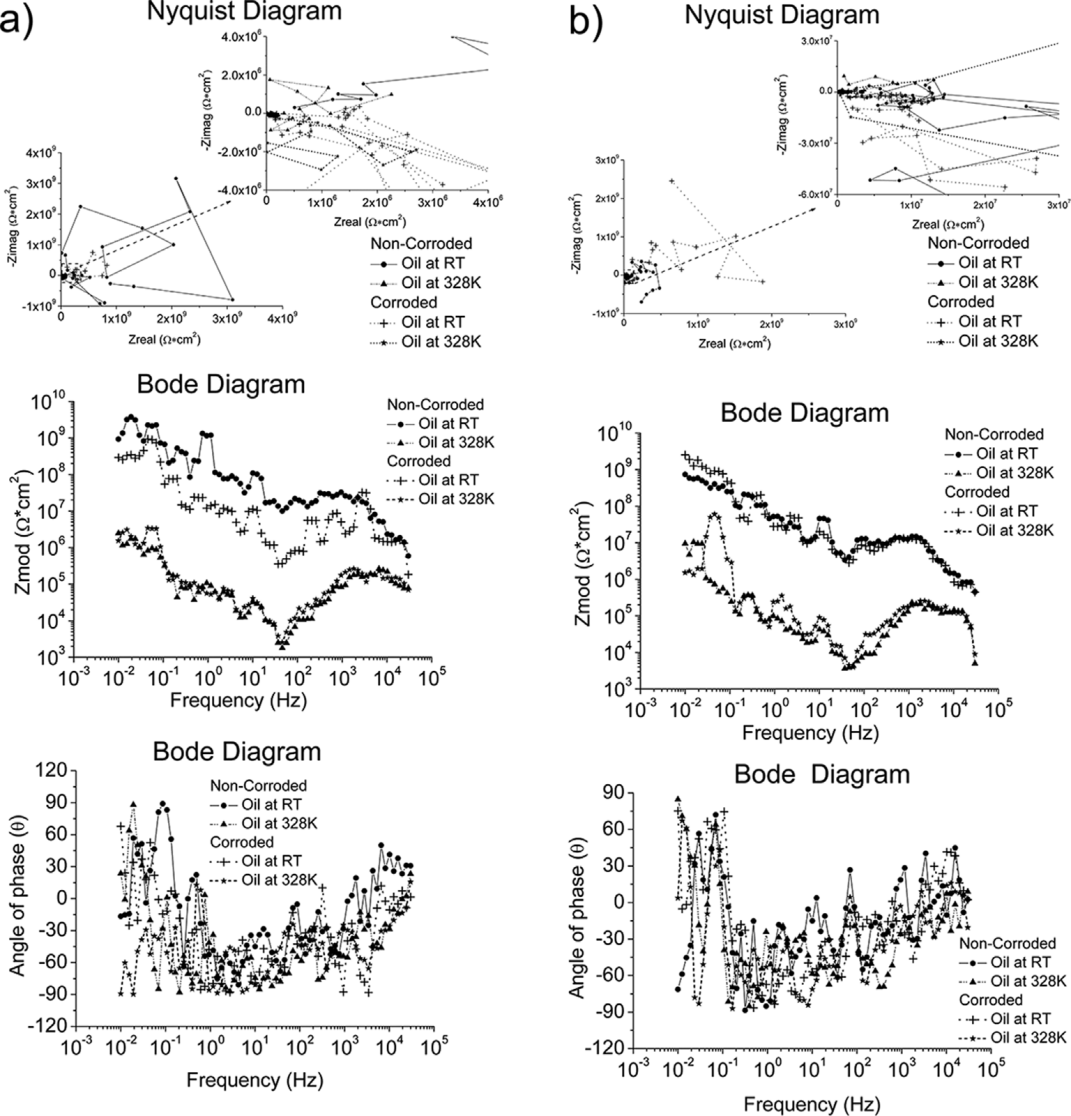

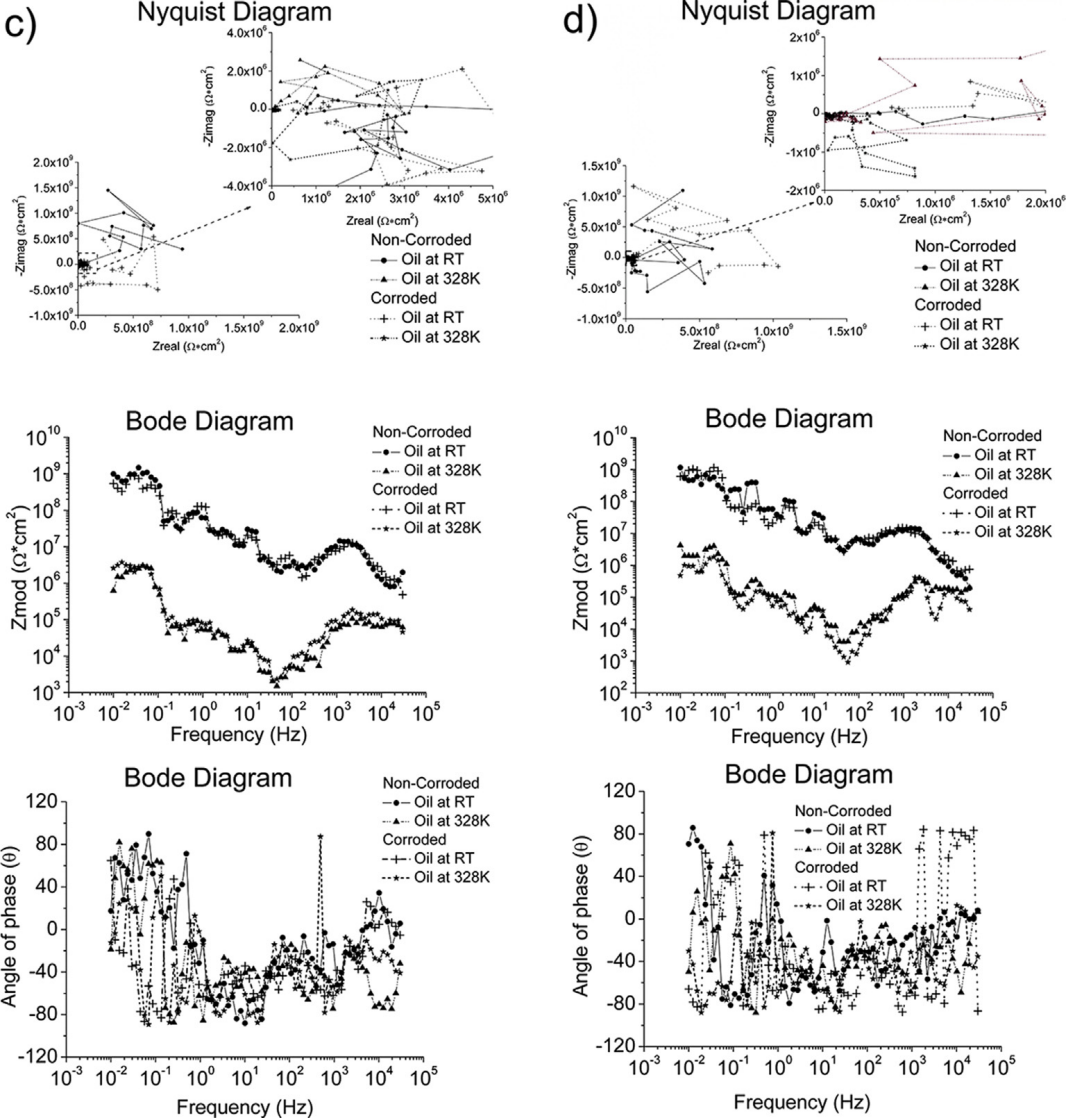
The Bode and Nyquist diagram of non-corroded and corroded materials immersed in oil at room temperature and at 328K: a) SS430, b) S235, c) AA1010 and d) AA6061. Note: The zoom-in area from the Nyquist diagram is shown in the insert plot.

Whilst ZRA and EIS were performed using the same test conditions in all environments, OCP and EIS were conducted using different test conditions depending on the environment. The test conditions used to generate the reported data are specified in Table 7. The complementary information in a format of Nyquist and Bode diagrams to represent the experimental raw data are also presented in Fig. 1, Fig. 2, Fig. 3, Fig. 4.

**Table 7:** Test conditions used in conjunction with the four electrochemical analysis techniques.

Environment	Electrochemical analysis techniques			
	OCP	ZRA	EIS	PPC
Seawater	*f*/*D.A*, 10Hz/0.1s *t*_*Total*_; 2 hours	*f*/*D.A*, 10Hz/0.1s *t*_*Total*_; 2 hours	Δ*f*; 0.01–30000Hz Points; 70 Point/decade; 10 Δ*V*_*(R.M.S*)_; 0.01V	*S.R*.; 1.67*10^−4^V/s *E*_*ini*_; *E*_*ocp*_-0.3V *E*_*final*_; 3V vs *E*_*ref*_ *I*_*lim*_; 0.01A/cm^2^
Grease at RT, pH = 5.2	*f*/*D.A*, 0.3Hz/3s *t*_*Total*_; 2 hours	–		*S.R*.; 5*10^−3^V/s *E*_*ini*_; *E*_*ocp*_-1V *E*_*final*_; 3V vs *E*_*ref*_ *I*_lim_;0.01A/cm^2^
Grease & Seawater at RT, pH = 4.3				
Grease & Seawater at 328K, pH = 6.8				
Oil at RT, pH = 8.8				
Oil at 328K, pH = 8.6
